# Ostéochondrite du condyle fémoral

**DOI:** 10.11604/pamj.2014.18.194.4870

**Published:** 2014-07-05

**Authors:** Younes Ouchrif, Issam Elouakili

**Affiliations:** 1 Service de Chirurgie Orthopédique, CHU, Rabat, Maroc

**Keywords:** Ostéochondrite, condyle fémoral, fracture, osteochondritis, femoral condyle, fracture

## Image en médecine

L'Ostéochondrite disséquante des condyles fémoraux est une affection rare. Elle se définit comme une zone localisée de modifications vasculaires atteignant l'os sous chondral. La physiopathologie n'est pas connue mais l'hypothèse mécanique et vasculaire reste au premier plan. La classification de Bedouelle permet une analyse précise de la lésion et a un intérêt dans l'indication thérapeutique. La symptomatologie est dominée par la douleur mécanique du genou, rarement une sensation d'accrochage ou de blocage lorsque le fragment est libre en intra articulaire. Le diagnostic est radiologique. L'arthroscanner a pour intérêt l’étude de la stabilité du fragment. L'IRM et la scintigraphie permettent l’étude de sa vitalité. Les techniques chirurgicales sont multiples et peuvent aller de la simple fixation du fragment jusqu’à des techniques de reconstructions cartilagineuses lorsque le fragment n'est pas viable et le défect cartilagineux est important. Le pronostic à long terme est dominé par la survenu d'une arthrose pouvant nécessiter la mise en place d'une prothèse totale du genou.

**Figure 1:**
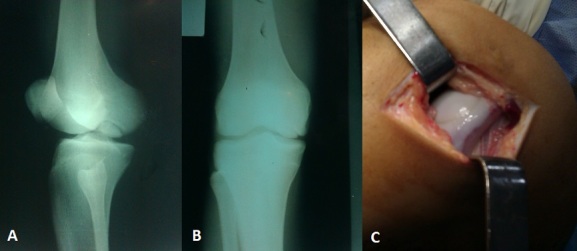
A) incidence de ¾ du genou montrant l'ostéochondrite du condyle fémoral, B) incidence de face montrant le fragment ostéochondral, C) vu per opératoire montrant le fragment ostéochondral

